# A New Provocative Test for Glaucoma

**DOI:** 10.5005/jp-journals-10008-1194

**Published:** 2016-05-12

**Authors:** Fabio N Kanadani, TCA Moreira, LF Campos, MP Vianello, J Corradi, SK Dorairaj, ALA Freitas, R Ritch

**Affiliations:** Chief, Department of Ophthalmology, Medical Science Ophthalmology Institute, Belo Horizonte, Brazil; Assistant Professor, Department of Retina, Medical Science Ophthalmology Institute, Belo Horizonte, Brazil; Assistant Professor, Department of Glaucoma, Medical Science Ophthalmology Institute, Belo Horizonte, Brazil; Assistant Professor, Department of Glaucoma, Medical Science Ophthalmology Institute, Belo Horizonte, Brazil; Assistant Professor, Department of Glaucoma, Medical Science Ophthalmology Institute, Belo Horizonte, Brazil; Assistant Professor, Department of Ophthalmology/Glaucoma, Mayo Clinic Jacksonville, Florida, USA; Assistant Professor, Department of Glaucoma, Medical Science Ophthalmology Institute, Belo Horizonte, Brazil; Assistant Professor, Department of Ophthalmology/Glaucoma, Mayo Clinic Jacksonville, Florida, USA

**Keywords:** Glaucoma, Provocative test, Water drinking test.

## Abstract

**Purpose:** To compare the effects of the water-drinking test (WDT) with the 30° inverted body position test on intraocular pressure (IOP) in normal patients, suspected glaucoma patients and glaucoma patients.

**Materials and methods:** Based on clinical evaluation of the optic disk, IOP, and standard achromatic perimetry (SAP) of 71 eyes, 18 were “normal” (normal SAP and optic disk evaluation, and IOP < 21 mm Hg), 30 were “glaucoma suspect” (GS; normal SAP, cup/disk (C/D) ratio > 0.5 or asymmetry > 0.2 and/or ocular hypertension), and 31 had “early glaucoma” (MD < -6 dB, glaucomatous optic neuropathy). Standard achromatic perimetry was performed with the Octopus 3.1.1 Dynamic 24-2 program. Patients fasted before the WDT, and four measurements were performed at basal, 15’, 30, and 45’ after drinking 1 liter of water (WDT) in 5 minutes. In the 30° inverted position, IOP measurement with Perkins applanation tonometer was taken after 5 minutes lying down.

**Results:** There was a statistical difference in all groups between the basal IOP and peak IOP during the WDT (p < 0.001) and in the inverted position IOP (p < 0.001). Controls (p = 0.50), suspects (p = 0.41) and glaucoma patients (p = 1.0) did not exhibit a difference between WDT-IOP and inverted position IOP.

**Conclusion:** The 30° inverted position test was as efficient as WDT in detecting peak IOP. This new provocative test is easier, faster and more comfortable for both patients and doctors.

**How to cite this article:** Kanadani FN, Moreira TCA, Campos LF, Vianello MP, Corradi J, Dorairaj SK, Freitas ALA, Ritch R. A New Provocative Test for Glaucoma. J Curr Glaucoma Pract 2016;10(1): 1-3.

## INTRODUCTION

Elevated intraocular pressure (IOP) is still considered the main risk factor for the development of glaucomatous optic neuropathy (GON). Target IOP is one at which no additional damage is expected to occur. The benefits of lowering IOP have already been demonstrated by numerous previous studies.^1,2^ However, a significant group of patients still develops glaucomatous progression despite IOP values considered within adequate limits.^3,4^

This could be explained by IOP fluctuation during the day or by pressure peaks not detected during office examinations. Drance^5^ demonstrated that almost one-third of patients with single IOP measurements at office hours had pressure peaks only detected during a 24-hour diurnal tension curve (DTC). Although this would be the best way to detect IOP peaks, DTC is not always feasible in routine practice.

Another possible way to assess the IOP is the water-drinking test (WDT). This test was first described in the 1960s as a diagnostic test for glaucoma. After water ingestion, a 6 or 8 mm Hg rise in IOP was considered a positive test. However, this test presented unacceptable false positive and false negative results.^6^ On the other hand, the WDT presents a good correlation between IOP peaks after water overload and IOP peaks detected during a daily tension curve. Also, the importance of this test was demonstrated by Armaly et al.^7^ In a prospective study of 5000 patients with open angle glaucoma, these authors found five potential risk factors for the development of glaucomatous visual field lesion: outflow facility, age, IOP, cup/disk ratio and change in IOP after water ingestion. All these data have changed the concept of the WDT, which is not used as a diagnostic test anymore, but as a useful tool to assess IOP peaks.

Although we have many provocative tests, none of them seems to be comfortable for the patients and doctors. In this study, we are going to describe a potential new provocative test for glaucoma. The 30° inverted body position is an easy and fast way to induce IOP rise.

## MATERIALS AND METHODS

In prospective comparative study, 79 patients were enrolled. All patients were recruited in the Santa Casa’s Eye Clinic of Belo Horizonte. The normal individuals were volunteers who were friends or parents of the suspects and glaucoma patients. Informed consent was obtained from all participants following the tenets of the declaration of Helsinki.

The inclusion criteria for an eye included: visual acuity ≥ 20/40, no clinical signs of macular disease, refractive error between ± 6 Diopters spherical and ± 3 Diopters cylindrical.

**Fig. 1:**
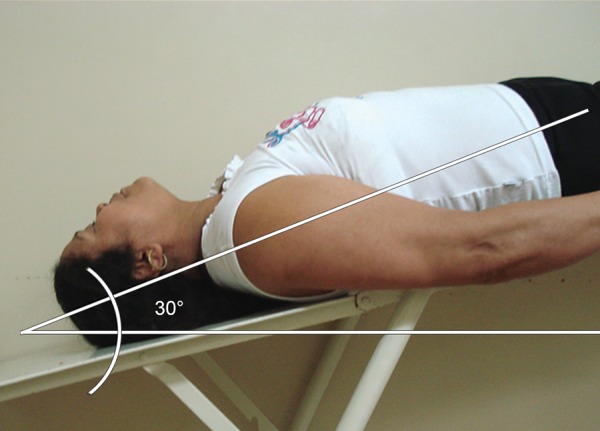
The 30° inverted body position test

Based on clinical evaluation of the optic disk, IOP, and standard achromatic perimetry (SAP) of 79 eyes, 18 were “normal” (normal SAP and optic disk evaluation, and IOP < 21 mm Hg), 30 were “glaucoma suspect” (GS; normal SAP, C/D ratio > 0.5 or asymmetry > 0.2 and/ or ocular hypertension), and 31 had “early glaucoma” (MD < -6 dB, glaucomatous optic neuropathy). Standard achromatic perimetry was performed with the Octopus 3.1.1 Dynamic 24-2 program. Patients fasted before water-drinking test, and four measurements were performed at basal, 15’, 30’ and 45’ after drinking 1 liter of water (WDT) in 5 minutes. In the 30° inverted position, IOP measurement with Perkins applanation tonometer was taken after 5 minutes lying down (Fig. 1).

The statistical analysis was performed with the Statistical Package for the Social Sciences (SPSS) 10.1 (SPSS Inc. Chicago, IL, EUA). Results were expressed as mean ± standard deviation and paired Student’s t-test was used to evaluate the level of significance. A p-value of 0.05 or less was considered significant. Two non-parametric tests were used to confirm the test above―Friedman and Wilcoxon.

## RESULTS

There was a statistical difference in all groups between the basal IOP and peak IOP during the WDT (p < 0.001) and in the inverted position IOP (p < 0.001). Controls (p = 0.50), suspects (p = 0.41) and glaucoma patients (p = 1.0) did not exhibit a difference between WDT-IOP and inverted position IOP (Tables 1 and 2).

**Table 1:** **Table 1:** Water-drinking test values

		*IOP (mm Hg) (Mean ± SD)*									
		*Basal*		*15’*		*30’*		*45’*		*Peak*	
Control		13.67		15.39		16.83		15.89		18.22	
		2.63		3.05		3.31		3.88		3.47	
Glaucoma suspects		15.59		18.00		19.41		18.06		19.93	
		4.19		4.06		4.15		4.54		3.69	
Glaucomatous		16.23		18.60		17.77		17.87		20.28	
		3.97		3.94		3.32		3.40		4.33	

**Table 2:** **Table 2:** Intraocular pressure peak comparison between WDT and 30° body inverted position test

		*IOP (mm Hg) (mean ± SD)*					
		*WDT peak*		*Inverted body* *position peak*		*p-value*	
Control		18.22		17.50		0.45	
		3.47		2.83			
Glaucoma suspects		19.93		20.67		0.49	
		3.69		4.50			
Glaucomatous		20.28		20.28		1.00	
		4.33		4.21			

**Table 3:** **Table 3:** Comparison of provocative tests with two different statistical analysis

		*Friedman’s test*				*Wilcoxon’s test*			
		*Chi-square*		*p-value*		*V*		*p-value*	
Control		2		0.1573		99.5		0.5549	
Glaucoma suspects		0.36		0.5485		123		0.2922	
Glaucomatous		0.125		0.7237		264.5		1	

When the other tests were used, we could not reject the hypothesis that the WDT and 30° inverted body position test were different (Table 3).

## DISCUSSION

The WDT is a provocative test that was widely used a few decades ago to help in the diagnosis of open angle glaucoma,^8,9^ but was found to be inadequate due to many false positive and false negative results in 10 year prospective studies. However, after some years, the emphasis on the value of this test has changed. As a result of the correlation with the diurnal tensional curve,^8^ the WDT has been proposed as an alternative method to monitor IOP.

The importance of IOP peaks in the development of glaucoma progression has been already reported by Zeimer et al^9^ and also by Martinez-Bello et al.^10^ In our study, we found a significant difference between basal and peak IOP in both tests, showing that both tests were able to cause IOP rise.

Recently, Brubaker^11^ proposed that the WDT could be used as an indirect measurement of outflow facility to compare the IOP responses of glaucoma eyes to different drugs. Drugs, such as prostaglandins improve the outflow facility and are expected to show less IOP variation secondary to water challenge. The presence of any filtration surgery should also be considered when comparing WDT results between eyes. Earlier studies already showed a relatively small range of diurnal IOP variation in trabeculectomized eyes.^12^

In the advanced glaucoma intervention study (AGIS), almost no visual field deterioration was observed in patients whose IOP was kept within safe levels.^13^ However, in a certain patients, it is possible to have glaucomatous progression despite apparently controlled IOP. The visual field loss could have occurred at a time when IOP is high, although it was controlled at the time of measurement. It has been suggested that large diurnal fluctuations in IOP^13-15^ may be an additional risk factor in patients with glaucoma.^7^ Furthermore, IOP peaks may not be recognized in a single office IOP measurement and may be responsible for additional visual field loss despite apparently controlled IOP.

Zeimer RC et al^9^ have shown that, in a population with a 30% prevalence of progressive loss of visual field, 75% of the patients with pressure peaks have progressive loss and 75% of those without pressure peaks do not have visual field progression. Another group^15^ has found that 29% of patients with a progressive visual field loss had IOP peaks, compared with 5% of patients with stable visual fields. In the same study, the IOP range was also considered a strong and independent risk factor in patients with glaucoma. Therefore, the office measurement of IOP may not be sufficient to assess the adequacy of treatment. Nevertheless, the diurnal IOP assessment (diurnal tension curve) demands the entire day, which is not always feasible in busy clinics.

In the present study, a new provocative test was employed to describe IOP elevation in Yoga practi-tioners.^16-18^ In these studies, the patients were asked to stay in an inverted position while IOP was measured. Sometimes the IOP level would rise to as high as 35 mm Hg within seconds. Our results showed that keeping the body position inverted 30° for 5 minutes elevated the IOP in normals, suspects and glaucoma patients. Another important finding was that the control group IOP rise was lower than the IOP rise in glaucoma patients. This suggests that normal individuals could have a better ocular hemodynamic control leading to a more stable IOP.

The 30° inverted position test was as efficient as WDT in detecting peak IOP. It is important to highlight that most of the provocative tests for glaucoma are not feasible in clinical practice; some are uncomfortable for patients and their doctors and others take a long time.
